# Dietary Restriction Promotes Vessel Maturation in a Mouse Astrocytoma

**DOI:** 10.1155/2012/264039

**Published:** 2011-12-27

**Authors:** Ivan Urits, Purna Mukherjee, Joshua Meidenbauer, Thomas N. Seyfried

**Affiliations:** Biology Department, Boston College, Chestnut Hill, MA 0246, USA

## Abstract

Mature vasculature contains an endothelial cell lining with a surrounding sheath of pericytes/vascular smooth muscle cells (VSMCs). Tumor vessels are immature and lack a pericyte sheath. Colocalization of vascular endothelial growth factor receptor 2 (VEGFR-2) and platelet-derived growth factor receptor beta (PDGF-R**β**) reduces pericyte ensheathment of tumor vessels. We found that a 30% dietary restriction (DR) enhanced vessel maturation in the mouse CT-2A astrocytoma. DR reduced microvessel density and VEGF expression in the astrocytoma, while increasing recruitment of pericytes, positive for alpha-smooth muscle actin (**α**-SMA). Moreover, DR reduced colocalization of VEGF-R2 and PDGF-R**β**, but did not reduce total PDGF-R**β** expression. These findings suggest that DR promoted vessel normalization by preventing VEGF-induced inhibition of the PDGF signaling axis in pericytes. DR appears to shift the tumor vasculature from a leaky immature state to a more mature state. We suggest that vessel normalization could improve delivery of therapeutic drugs to brain tumors.

## 1. Introduction

Tumor vascularization, vital to neoplastic progression, provides nutrients and oxygen to the tumor [1–3]. Proliferation of vessel-forming endothelial cells is a limiting factor for tumor growth [4–7]. Accordingly, targeting tumor vessel proliferation decreases blood flow and nutrient availability, thus slowing tumor growth [8]. Tumors induce the proliferative vascular response of host blood vessels by influencing the local balance of angiogenic regulators, a rate-limiting step termed the angiogenic switch [9, 10]. The uncontrolled production of angiogenic stimulators and the absence of inhibitors favor vessel growth [10–12].

 Normal tissue vasculature contains an endothelial lining with a surrounding sheath of pericytes/vascular smooth muscle cells (VSMCs) [13]. In contrast to healthy vessels, tumor vessels are immature, often mal-shaped, irregular, and have a tortuous structure with a leaky endothelial cell lining [13, 14]. The process of blood vessel maturation involves ensheathment of neovascular sprouts by *α*-smooth-muscle-actin- (*α*-SMA-) positive pericytes [15]. Pericytes contact endothelial cells and play an active role in endothelial cell function and blood flow regulation [15–17]. Mature vessels contain a variety of contractile proteins including *α*-SMA, which is often used as a pericyte marker [15, 18, 19].

 The instability of tumor blood vessels is associated with the absence of a smooth muscle cell sheath [11]. Abnormalities in tumor vessel shape and structure not only impair drug delivery, but also can facilitate metastatic spread [20, 21]. While it may seem that an increase in blood vessel quantity would provide sufficient oxygen to tumors, the abnormal vessels deliver less oxygen leading to a hypoxic tumor environment [13]. This will further stimulate tumor growth and aberrant angiogenesis [22, 23]. Vascular endothelial growth factor (VEGF) and platelet-derived growth factor (PDGF) signaling drives angiogenesis and recruitment of perivascular cells to surround the newly formed blood vessels [24]. VEGF stimulates endothelial cell migration, proliferation, survival, permeability, and lumen formation and has become a prime target of antiangiogenic therapy [13]. Blockage of VEGF signaling induces vessel normalization and inhibition of new vessel growth (16). In addition to the pruning of immature blood vessels, inhibition of VEGF expression also increases pericyte cell coverage and vessel maturation [25, 26].

 Platelet-derived growth factor (PDGF) coordinates pericyte coverage of vascular sprouts through PDGF-R*β* on vascular smooth muscle cells [27]. Greenberg et al. showed that, in addition to stimulating endothelial cell proliferation, VEGF also inhibits neovascularization via its capacity to disrupt vascular smooth muscle cell function [24]. Specifically, VEGF prevents pericyte coverage of nascent vascular sprouts leading to vessel destabilization. VEGF activation of VEGF-R2 suppresses PDGF-R*β* signaling in VSMCs through the assembly of a complex consisting of the two receptors. Inhibition of VEGF-R2 prevents the formation of this receptor complex and restores tissue angiogenesis. Moreover, genetic deletion of tumor cell VEGF also disrupts the receptor complex and consequently increases tumor vessel maturation. These findings are important as they reveal a dichotomous role for VEGF signaling as a promoter of endothelial cell function and as an inhibitor of VSMCs and vessel maturation [24, 26, 28, 29].

 VEGF expression is greater in tumor cells than in normal cells [30–33]. Reduced VEGF expression reduces angiogenesis while increasing vessel maturation [24]. Mukherjee et al. demonstrated that a 30% dietary restriction (DR) inhibits angiogenesis and reduces prostate tumor growth [34]. We showed that DR in mice reduces microvessel density in experimental mouse and human brain tumors [35, 36]. Powolny et al. demonstrated that DR attenuates tumor growth and reduces vascular density. They also found that a 40% DR significantly reduced VEGF gene and protein expression in rat prostate tumors [37]. These studies show that DR is a potentially viable nontoxic therapeutic approach for managing malignant brain tumor growth, for reducing tumor angiogenesis, and for increasing long-term survival in mice bearing orthotopically implanted tumors [35–38].

 DR is produced by restricting the total caloric content administered to subjects. However, a distinction from starvation is that DR does not cause anorexia or malnutrition [34, 39–42]. It is important to note that the total reduction of calories, rather than the macronutritional content of the food, proves most important to producing the effects of reducing tumor growth and in limiting angiogenesis [34, 39]. Although prior studies showed that dietary restriction is antiangiogenic when initiated early in tumor development, no prior studies have identified the mechanisms by which dietary restriction is effective in correcting vasculature.

 In this paper, we show that DR enhances vessel maturation and stabilization in the highly vascularized CT-2A mouse astrocytoma. In addition to reducing VEGF expression, we also found that DR decreased colocalization of VEGF-R2 with PDGF-R*β*. Our findings suggest that DR imparts its antiangiogenic and vessel maturating effects on the CT-2A tumor via the reduction of VEGF expression promoting VSMC ensheathment of vascular sprouts.

## 2. Materials and Methods

### 2.1. Mice

Mice of the C57BL/6J strain were obtained from the Jackson laboratory (Bar Harbor, ME, USA). The mice were propagated in the animal care facility of the Biology Department of Boston College, using animal husbandry conditions described previously [43]. Male mice (8–10 weeks of age) were used for the studies and were provided with food either ad libitum (AL) or under restricted conditions (as described below). Water was provided ad libitum to all mice. The animal room was maintained at 22°C, and cotton nesting pads were provided for additional warmth. All animal experiments were carried out with ethical committee approval in accordance with the National Institutes of Health Guide for the Care and Use of Laboratory Animals and were approved by the Institutional Care Committee. 

### 2.2. Brain Tumor Model

The syngeneic CT-2A experimental mouse brain tumor was generated in our laboratory after implantation of 20-methylcholanthrene into the cerebral cortex of a C57BL/6 mouse according to the procedure of Zimmerman [45, 46]. Histologically, the CT-2A brain tumor is broadly classified as a poorly differentiated highly malignant anaplastic astrocytoma [46]. The tumor grows orthotopically as a soft, noncohesive, and highly vascularised mass.

### 2.3. Intracerebral Tumor Implantation

The CT-2A tumor was implanted into the cerebral cortex of C57BL/6J mice using a trocar as we previously described [47, 48]. Briefly, mice were anaesthetized with pentobarbital (Vet Labs, Inc) intraperitoneally and their heads were shaved and swabbed with 70% ethyl alcohol under sterile conditions. Small CT-2A tumor pieces (1 mm^3^) from a C57BL/6J donor mouse were implanted into the right cerebral hemisphere of anaesthetized recipient mice as we recently described [48]. All of the mice recovered from the surgical procedure and were returned to their cages when fully active. Initiation of tumors from intact tumor pieces is preferable to initiation from cultured cells since the pieces already contain an established microenvironment that facilitates tumor growth.

### 2.4. Dietary Restriction (DR)

The mice were separated prior to the beginning of the experiment and randomly assigned to either control group that was fed AL or to an experimental group that was fed a total dietary restriction (DR) of 30%. Each mouse was housed singly in a plastic shoe box cage with a filter top and was given a cotton nesting pad for warmth. DR was initiated 2 days following tumor implantation and was continued for 11 days following implantation. Total DR maintains a constant ratio of nutrients to energy; that is, the average daily food intake (grams) for the AL fed mice was determined every other day and the DR-fed mice were given 70% of that quantity on a daily basis. All mice received PROLAB chow (Agaway Inc.), which contains a balance of mouse nutritional ingredients and, according to the manufacturer's specification, delivers 4.4 Kcal/g gross energy. Body weights of all mice were recorded every other day. 

### 2.5. Tumor Growth and Histology

Intracerebral tumor growth was analyzed directly by measuring total tumor wet weight. Tumors were dissected from normal-appearing brain tissue, were frozen, and then were weighed. Tumor samples for histology were fixed in 10% neutral buffered formalin (Sigma) and embedded in paraffin. They were sectioned at 5 *μ*m, stained with haematoxylin and eosin, and examined by light microscopy. 

### 2.6. Measurement of Plasma Glucose and Lipids

Mice were anesthetized with isoflurane (Halocarbon Laboratories, River Edge, NJ, USA) and euthanized by exsanguination, involving collection of blood from the heart in heparinized tubes. The blood was centrifuged at 6,000 g for 10 min, the plasma was collected, and aliquots were stored at −80°C until analysis. Plasma glucose concentration was measured in a spectrophotometer using the Stanbio Enzymatic Glucose Procedure (Stanbio). High-performance thin-layer chromatography was used to evaluate plasma lipids according to our standard procedures [49].

### 2.7. Western Blot Analysis

Frozen CT-2A tumor and contralateral normal brain tissues were homogenized in lysis buffer, on ice. Lysates were transferred to Eppendorf tubes, mixed on a rocker for 1 h at 4°C, and then centrifuged for 20 min. Supernatants were collected, and protein concentrations were estimated using the Bio-Rad detergent-compatible protein assay. Approximately 25 *μ*g of total protein from each tissue sample were denatured with SDS-PAGE sample buffer and resolved with SDS-PAGE on 4% to 12% Bis-Tris gels (Invitrogen). Proteins were transferred to a polyvinylidene difluoride immobilon TM-P membrane (Millipore) overnight at 4°C and blocked in 5% nonfat powdered milk in TBS with tween 20 (pH 7.6) for 1 h at room temperature. Membranes were probed with primary antibodies overnight at 4°C with gentle shaking. The blots were then incubated with the appropriate secondary antibody for 1 h at room temperature, and bands were visualized with chemiluminescence. Each membrane was stripped and reprobed for *β*-actin as an internal loading control, and the ratio of the indicated protein to *β*-actin was analyzed by scanning densitometry.

### 2.8. Antibodies and Reagents

Antibodies were obtained against *α*-SMA (Sigma), *β*-Actin (Cell Signaling), Factor VIII (Dako), PDGF-R*β* (Santa Cruz), VEGF-R2 (Santa Cruz), and VEGF (Santa Cruz).

### 2.9. Confocal Microscopy

For the immunohistochemical studies, the tissue sections were deparaffinized, rehydrated, and washed. The tissue sections were then heat treated (95°C) in antigen unmasking solution (Vector Laboratories, Burlingame, CA, USA) for 30 min. Tissue sections were blocked in goat serum (1 : 10 in PBS) for 1 h at room temperature, treated with primary antibody, followed by treatment with secondary antibody. Corresponding tissue sections without primary antibody served as negative controls. For confocal microscopy, digital images were obtained on a Leica DMI6000 inverted scope equipped with the Leica TCSSP5 confocal system, using HCX PL APO 409/1.25 NA oil and HCX PL APO 639/1.4 NA oil objective lenses. Leica confocal software was used to acquire images.

 For *α*-SMA and Factor VIII immunoflourscent staining, sections were incubated with a cocktail of *α*-SMA and Factor VIII primary antibodies (1 : 100) in blocking buffer for 1 h at room temperature, followed by a cocktail of alexafluor 585 and 488, conjugated anti-mouse and anti-rabbit, respectively, secondary antibody (1 : 200) for 45 min at room temperature.

 For colocalization of VEGF-R2 and PDGF-R*β*, sections were incubated with a cocktail of VEGF-R2 and PDGF-R2 primary antibodies (1 : 100) in blocking buffer overnight at 4°C, followed by a cocktail of alexafluor 585 and 488, conjugated anti-mouse and anti-rabbit, respectively, secondary antibody at 1 : 200 dilution for 45 min. All other conditions were as stated. 

### 2.10. Immunohistochemistry

Tissue sections were processed similarly as for confocal microscopy. Sections were treated with VEGF, PDGF-R*β*, and Factor VIII primary antibodies overnight at 4°C followed by treatment with a biotinylated anti-rat secondary antibody at 1 : 100 dilution (Vector laboratories, Inc). The sections were then treated with avidin biotin complex followed by 3,3-diaminobenzidine as substrate for staining according to the manufacturer's protocol (Vectastain Elite ABC kit, Vector laboratories, Inc.). The sections were counterstained with haematoxylin and mounted. Corresponding tissue sections without primary antibody served as negative controls. The Zeiss Axioplan 2 light microscope was used to capture bright-field images.

## 3. Results

### 3.1. Dietary Restriction Reduces Body Weight, Blood Glucose, and Intracerebral Tumor Growth

The DR group exhibited an average body weight reduction of 22 ± 1% and an average tumor reduction of 76 ± 4%. Average tumor wet weight was significantly lower in the DR-fed mice (51 ± 7 mg) than in the AL-fed mice (209 ± 40 mg); *P* < 0.01, Student's *t*-test. Blood glucose levels (mmol/L) in the AL and DR mice were 8.6 ± 0.6 and 4.4 ± 1.0, respectively (*P* < 0.05, determined by two-tailed *t*-test). The blood glucose levels were reduced in the DR mice as we previously showed [36, 50]. No significant differences were detected between the AL and DR mice for the distribution of major lipids including cholesteryl esters, cholesterol, triglycerides, or phosphatidylcholine (data not shown). Mouse activity level increased under dietary energy restriction. This is a well-documented phenomenon that occurs in all mice when placed under calorie restriction and is due to increased foraging [51]. It is difficult to determine if increased physical activity causes psychological stress. It is well documented that general health and fitness improves significantly in mice when they are underfed with adequate nutrition [51, 52]. It is important to note that all tumors implanted grew in both the DR and AL groups, indicating that dietary restriction did not inhibit tumor take. This study confirms previous observations that DR inhibits CT-2A tumor growth [36].

### 3.2. Dietary Restriction Reduces Microvessel Density and Hemorrhaging in the CT-2A Astrocytoma

**Figure 1:**
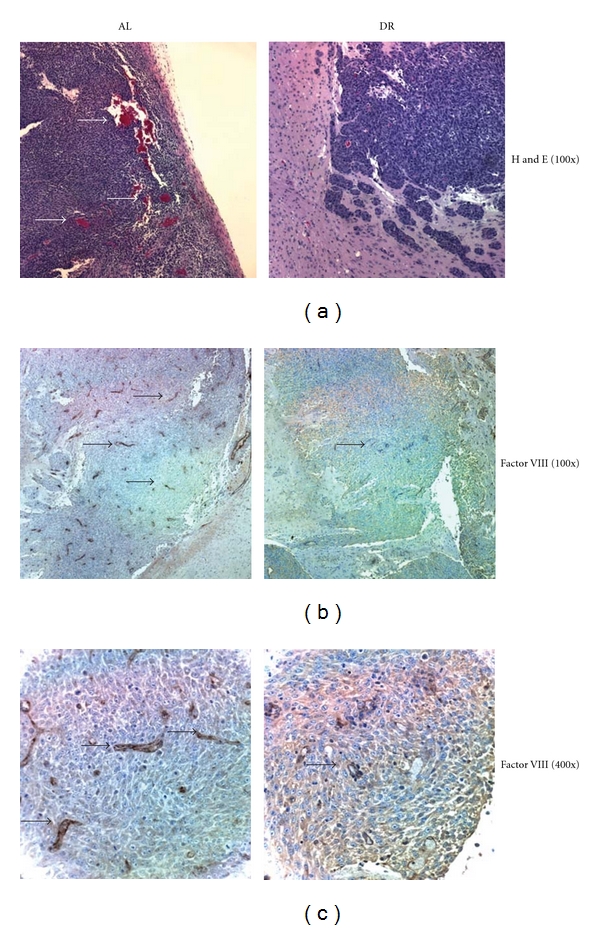
Dietary restriction reduces microvessel density and hemorrhaging in the CT-2A astrocytoma. (a) Vessel morphology. Arrows indicate hemorrhagenic regions. (b) Microvessel density. (c) Higher magnification. Arrows indicate positive Factor VIII vessel staining. Each stained section was representative of the entire tumor. All images were produced from digital photography.

H&E staining was used to evaluate the influence of DR on hemorrhagic blood vessels in CT-2A (Figure 1(a)). Light pink staining indicates normal brain tissue. Bright pink staining found within the CT-2A tumor tissue indicates hemorrhagic vasculature. The number of hemorrhagic vessels was noticeably less in tumors of DR-fed mice than in tumors of AL-fed mice. Factor VIII immunohistochemistry was used to evaluate the influence of DR on the density of vascular endothelial cells. The number of Factor-VIII-stained endothelial cells was noticeably less in sections of tumors from DR-fed mice than from AL-fed mice, indicating a reduction of microvessel density (Figures 1(b) and 1(c)).

### 3.3. Dietary Restriction Increases Maturation of Blood Vessels in the CT-2A Astrocytoma

**Figure 2:**
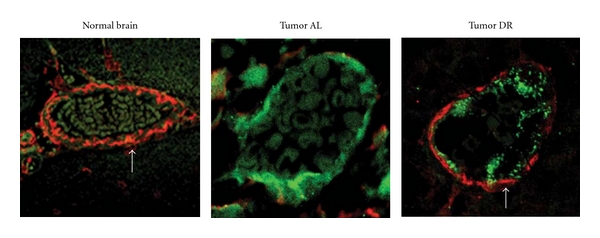
Influence of dietary restriction on blood vessel maturation in the CT-2A astrocytoma. Confocal analysis of normal brain and CT-2A tumor tissue double stained for *α*-SMA in vascular smooth muscle cells (red) and Factor VIII in vascular endothelial cells (green). Results show that *α*-SMA is greater in the vessels of the DR-fed tumor than in the vessels of the AL-fed tumor (indicated by white arrow). A blood vessel of a normal brain is shown for comparison. All other conditions were as described in Section 2.

Confocal microscopy was used to determine the influence of DR on localization of *α*-SMA and Factor VIII in blood vessels of CT-2A (Figure 2). *α*-SMA (red) was used as a marker for vascular smooth muscle cell (VSMC)/perictye coverage of blood vessels, and Factor VIII (green) was used as a marker for endothelial cells. Localization of VSMCs (red) with the endothelial cell lining (green) was greater in tumor vessels of DR-fed mice than in tumor vessels of AL-fed mice. These findings suggest that DR enhances VSMC/perictye coverage of CT-2A tumor vessels.

### 3.4. Dietary Restriction Increases *α*-SMA Expression and Reduces Factor VIII Expression in the CT-2A Astrocytoma

**Figure 3:**
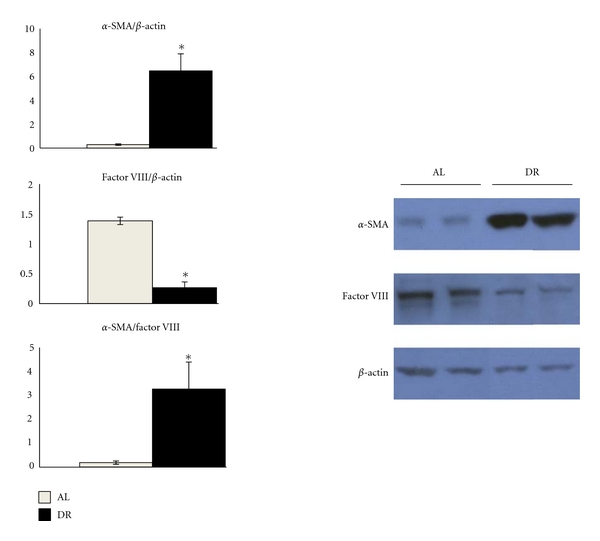
Dietary restriction increases *α*-SMA and reduces Factor VIII expression in the CT-2A astrocytoma. The histograms show the average relative expression of the indicated protein normalized to *β*-actin based on Western blot analysis. Equal amounts of protein were loaded into each lane of the Western blot (25 *μ*g). Other conditions were as described in Section 2. Values are expressed as normalized means of three to four independent tissue samples per group ± SEM. The value is significantly different in the tumors of DR-fed mice than in the tumors of AL-fed mice: *P < 0.05, Student's *t*-test. Two representative samples are shown for each tissue type.

Western blot analysis was done to examine the effects of DR on the relative expression of *α*-SMA and Factor VIII in tumor blood vessels. Factor VIII expression was significantly lower while *α*-SMA expression was significantly higher in the CT-2A tumor when grown in DR-fed mice than when grown in AL-fed mice (Figure 3). The ratio of *α*-SMA to Factor VIII was significantly greater in the tumors of the DR mice as compared to the AL mice (Figure 3). An increase of this ratio in DR mice as compared to the AL group indicates a reduction of endothelial cell proliferation and a simultaneous increase of pericyte/VSMC vessel coverage.

### 3.5. Dietary Restriction Reduces VEGF Expression in the CT-2A Astrocytoma

**Figure 4:**
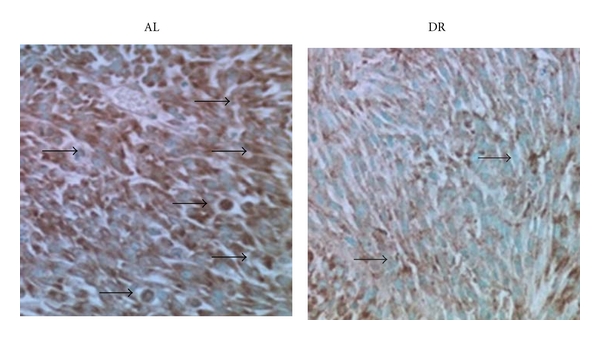
Dietary restriction reduces VEGF expression in the CT-2A astrocytoma. VEGF immunostained sections (400x). Results show that VEGF expression (brown stain) is less in the tumors of DR-fed mice than in the tumors of AL-fed mice. Black arrows indicate positive VEGF staining. Each stained section is representative of the entire tumor. All other conditions are as described in Section 2.

Immunohistochemistry was used to determine the influence of dietary restriction on local VEGF expression in CT-2A. The brown VEGF staining intensity and quantity was noticeably less in tumors of DR-fed mice than in tumors of AL-fed mice (Figure 4). These findings are consistent with previous findings in plasma indicating that DR reduces VEGF expression [35, 36].

### 3.6. Dietary Restriction Reduces PDGF-R*β* and VEGF-R2 Association in the CT-2A Astrocytoma

**Figure 5:**
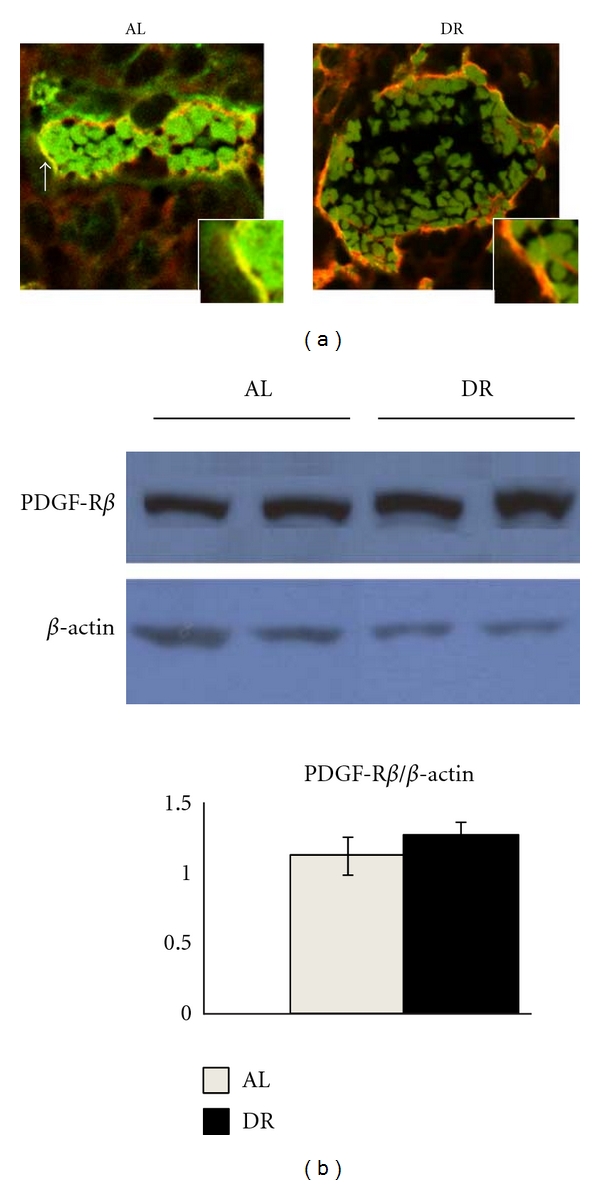
Dietary restriction reduces PDGF-R*β* and VEGF-R2 association in the CT-2A astrocytoma. (a) Confocal analysis of CT-2A tumor tissue double stained for VEGF-R2 (green) and PDGF-R*β* (red). Results show that the colocolization (yellow) of VEGF-R2 and PDGF-R*β* is less in the tumors of DR-fed mice than in the tumors of AL-fed mice. White arrow indicates colocolization. All other conditions are as described in Section 2. (b) The histogram shows that the average relative expression of PDGF-R*β* normalized to *β*-actin based on Western blot analysis is similar in the tumors of DR-fed and AL-fed mice. Equal amounts of protein were loaded into each lane (25 *μ*g), and the other conditions were as described in Section 2. Values are expressed as normalized means of three to four independent tissue samples per group ± SEM. There is no significant difference between values of DR-fed and AL-fed mice, Student's *t-*test. Two representative samples are shown for each tissue type.

Confocal microscopy showed that the amount of yellow staining, indicative of colocalization of PDGF-R*β* (red) and VEGF-R2 (green), was less in DR-fed mice compared to tumors of AL-fed mice (Figure 5(a)). Western blot analysis showed that PDGF-R*β* expression was similar in tumors of DR-fed and AL-fed mice (Figure 5(b)).

## 4. Discussion

We found for the first time that DR could enhance tumor blood vessel maturation in a malignant mouse astrocytoma. DR not only curtailed angiogenesis, but also increased vessel pericyte ensheathment in the highly vascularized CT-2A astrocytoma. DR reduced endothelial cell proliferation, as indicated by a reduction in staining for Factor VIII, a marker for endothelial cells. We also observed an increase of pericyte and vascular smooth muscle cell coverage of the endothelial cell lining in blood vessels, as indicated by an increase of *α*-SMA, a marker for VSMCs and VSMC-like pericytes. This was apparent from the increased ratio of *α*-SMA relative to Factor VIII. Our findings agree with the previously documented antiangiogenic effects of DR [34–36, 53]. Immunostaining of tumor sections with VEGF antibody showed an overall reduction of VEGF expression in DR-treated tumors. We suggest that the observed reduction in VEGF leads to the antiangiogenic and vessel maturating effects, via the VEGF-VEGF-R2 signaling axis.

 The VEGF-VEGF-R2 signaling axis is a primary pathway in endothelial cell proliferation [54]. Moreover, Greenberg et al. implicated VEGF in a dichotomous role. Apart from acting as a promoter of endothelial cell function, VEGF also acts as a negative regulator of VSMCs and consequently vessel maturation [24]. We observed that DR reduced colocalization of VEGF-R2 and PDGF-R*β* in the CT-2A tumor. We also found that DR had no significant influence on PDGF-R*β* expression. These findings suggest that DR blocks the association of VEGF-R2 and PDGF-R*β* by reducing VEGF, thus preventing inhibition of the PDGF signaling axis due to colocolization of the two receptors [24]. Further studies will be needed to evaluate these signaling pathways.

 An immature and leaky vasculature is a hallmark of solid tumors [55–57]. The leakiness of the tumor vasculature leads to elevated interstitial fluid pressure within the tumor. Tong et al. demonstrated that increased hydrostatic pressure within tumors hinders drug penetration across tumor vessels [58]. They also showed that penetration of large molecules into tumors is better through vessels with uniform pericyte coverage than through vessels with irregular pericyte coverage. We suggest that DR may improve drug delivery to the tumor via a similar mechanism. Denny et al. found that restriction of a ketogenic diet improved delivery of a small drug molecule into the mouse brain. They found that brain N-butyldeoxynojirmycin (NB-DNJ) content was 3.5-fold greater in the restricted ketogenic diet + NB-DNJ mice than in the NB-DNJ group alone suggesting that DR enhances delivery of the drug to the brain. This could allow for a lower dosing to achieve therapeutic effect [59]. Further research on the effects of dietary restriction on the brain vasculature is necessary to elucidate the mechanism by which diet and DR enhance drug delivery to the brain.

 It is well documented that prognosis is poor for most patients with malignant glioma [60]. The targeting of angiogenesis has become an important strategy in current therapy [61, 62]. The goal of antiangiogenic therapy is to reduce microvessel density and to increase vessel stabilization [9, 60–62]. We suggest that DR normalizes tumor vasculature by decreasing VEGF expression in the tumor. Our findings show that mature smooth-muscle-cell-covered vessels are more prominent in brain tumors under DR than in brain tumors under AL feeding.

## 5. Conclusions

The results indicate that dietary restriction promotes vessel maturation in an experimental mouse astrocytoma. This preclinical study shows that DR may be an effective nontoxic antiangiogenic therapy in brain tumors. DR may also improve drug delivery to brain tumors and may therefore be used in conjunction with drug therapy.

## Abbreviations

CT-2A: Mouse astrocytoma
DR: Dietary restriction
AL: Ad libitum
*α*-SMA: Alpha smooth muscle actin
VSMC: Vascular smooth muscle cell
VEGF: Vascular endothelial growth factor
VEGF-R2: Vascular endothelial growth factor receptor 2
PDGF-R*β*: Platelet-derived growth factor receptor *β*.
